# Supplementary material for: Culex Mosquito Piwi4 Is Antiviral against Two Negative-Sense RNA Viruses
Source: Viruses. 2022 Dec 10;14(12):2758. doi: 10.3390/v14122758 (PMC9781653; doi:10.3390/v14122758)
Supplement: Supplementary file 1 [file viruses-14-02758-s001.zip › viruses-2010696-supplementary.pdf]

Supplementary Table S1. *Aedes aegypti*, *Drosophila melanogaster*, *Culex quinquefasciatus*, and *Culex tarsalis* PIWI genes used for phylogenetic tree analysis.

Species	Gene	Accession Number
Aedes aegypti	AePiwi1	AAEL008076
	AePiwi2	AAEL008098
	AePiwi3	AAEL013692
	AePiwi4	AAEL007698
	AePiwi5	AAEL013233
	AePiwi6	AAEL013227
	AePiwi7	AAEL006287.1
	AeAgo3	AAEL007823
	AeZuc	AAEL011385
Drosophila melanogaster	DmPiwi	NM_057527
	DmAgo3	EF211827
	DmZuc	NM_135686.3
Culex quinquefasciatus	CqPiwi1	XM_001844015.2
	CqPiwi3	XM_038255983.1
	CqPiwi4	XM_038251629.1
	CqPiwi5	XM_038251859.1
	CqPiwi6a	XM_038251857.1
	CqPiwi6b	XM_038258148.1
	CqPiwi7	XM_038266499.1
	CqZuc	XM_001870711.2
	CqAgo3	XM_038254040.1
Culex tarsalis	CtPiwi1	mRNA6102*
	CtPiwi4	mRNA2582*
	CtPiwi5	mRNA13996*
	CtPiwi6	mRNA4897*
	CtPiwi7	mRNA5433*
	CtZuc	mRNA10624*
	CtAgo3	mRNA4166*

**Internal identifier – sequences are available in Supplemental Material S1.*

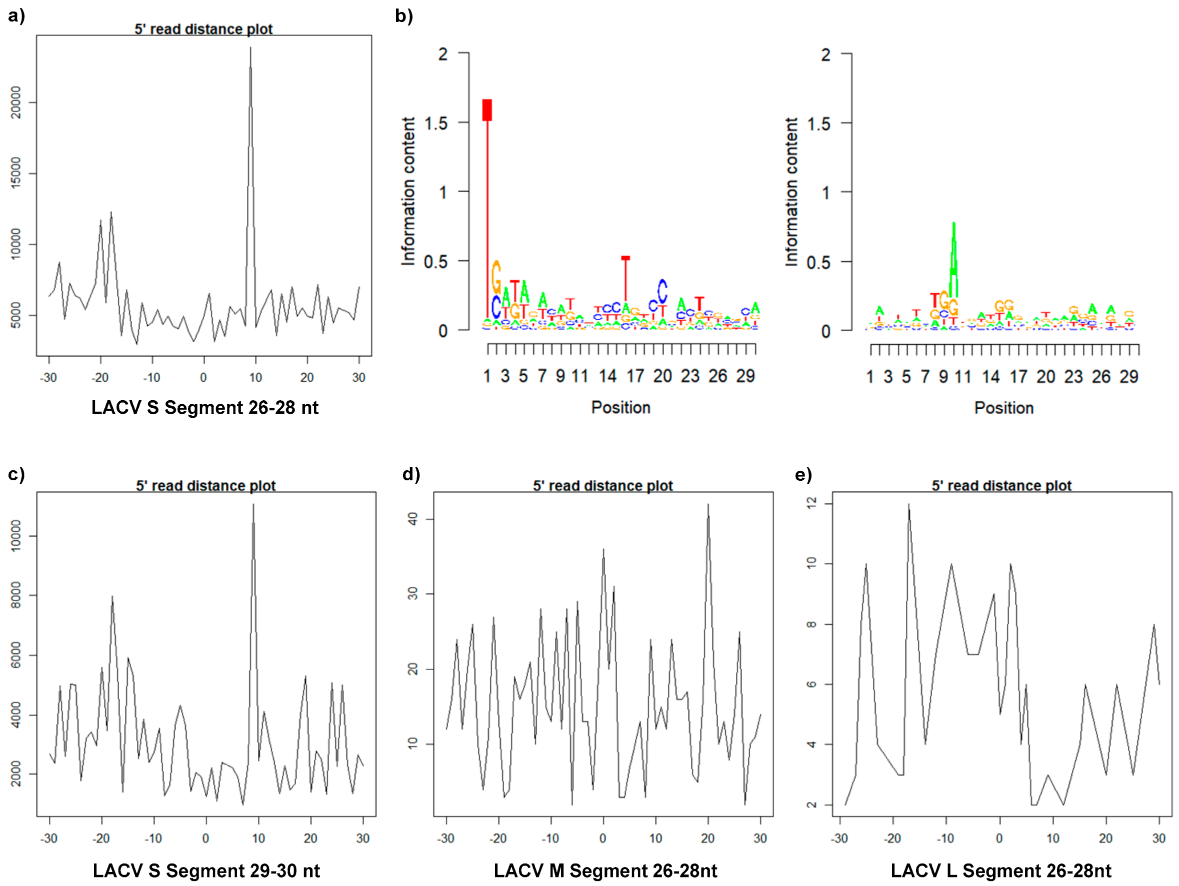

Supplemental Figure S1. Virus-derived sRNA reads in *Cx. quinquefasciatus* cells. sRNA libraries were sequenced from Hsu cells 2 dpi with LACV and aligned to the S segment (a, b, c), LACV M segment (d), LACV L segment (e). Distance plots were generated from LACV-derived 26-28 nt sRNA reads for the LACV S (a), LACV M (d), and LACV L (e) segment, or LACV derived 29-30 nt sRNA reads for the LACV S segment (c). Nucleotide biases of 29-30 nt sRNA reads derived from antisense (left) and sense (right) for LACV S segment are also shown (b).

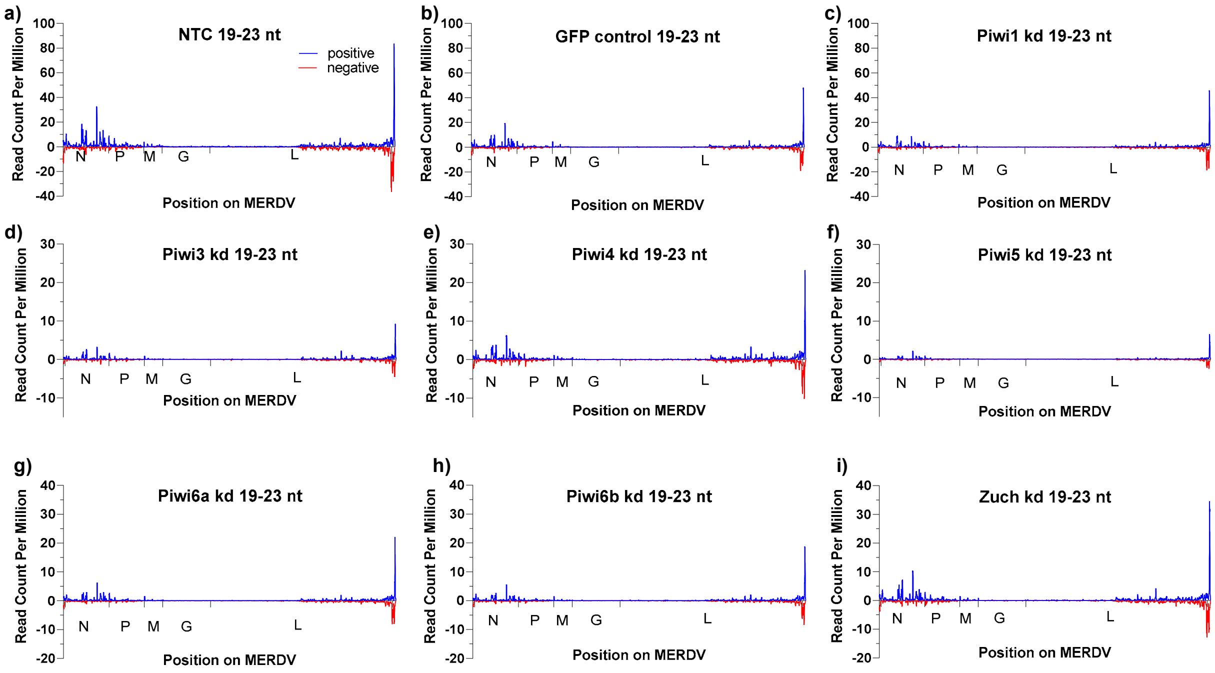

Supplemental Figure S2. 19-23 nt sRNA reads mapped to MERDV genome. Hsu cells were treated with either no dsRNA (a), GFP dsRNA (b), Piwi1 dsRNA (c), Piwi3 dsRNA (d), Piwi4 dsRNA (e), Piwi5 dsRNA (f), Piwi6a dsRNA (g), Piwi6b dsRNA (h), or Zuc dsRNA (i).

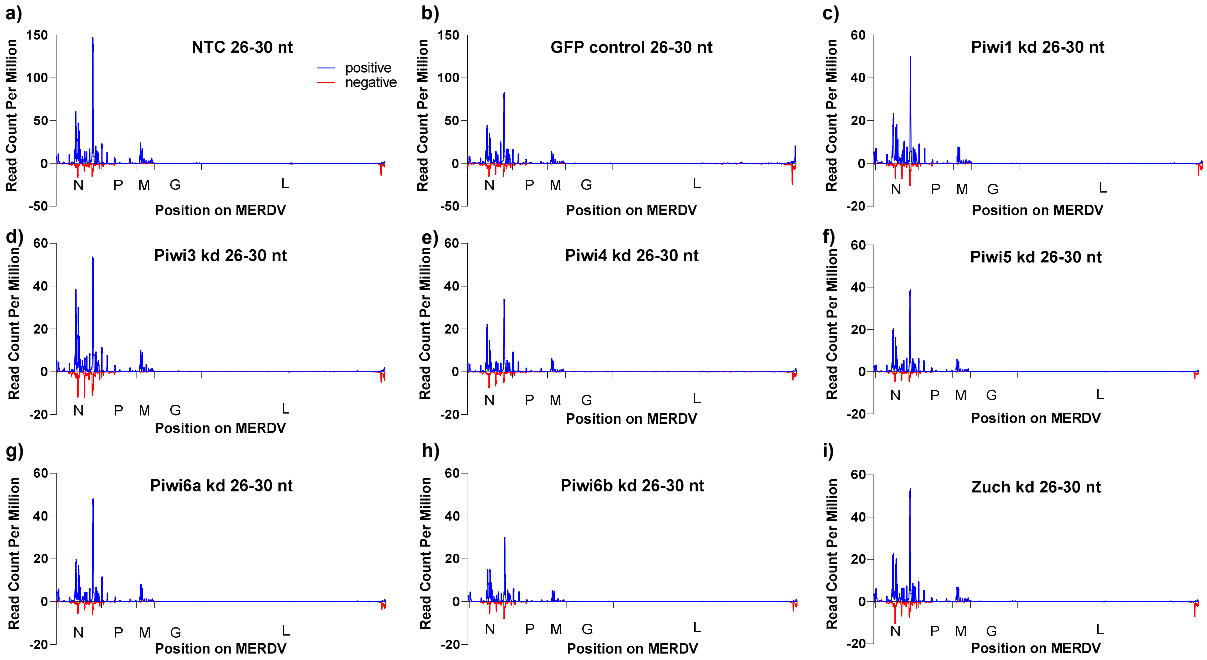

Supplemental Figure S3. 26-30 nt sRNA reads mapped to MERDV genome. Hsu cells were treated with either no dsRNA (a), GFP dsRNA (b), Piwi1 dsRNA (c), Piwi3 dsRNA (d), Piwi4 dsRNA (e), Piwi5 dsRNA (f), Piwi6a dsRNA (g), Piwi6b dsRNA (h), or Zuc dsRNA (i).

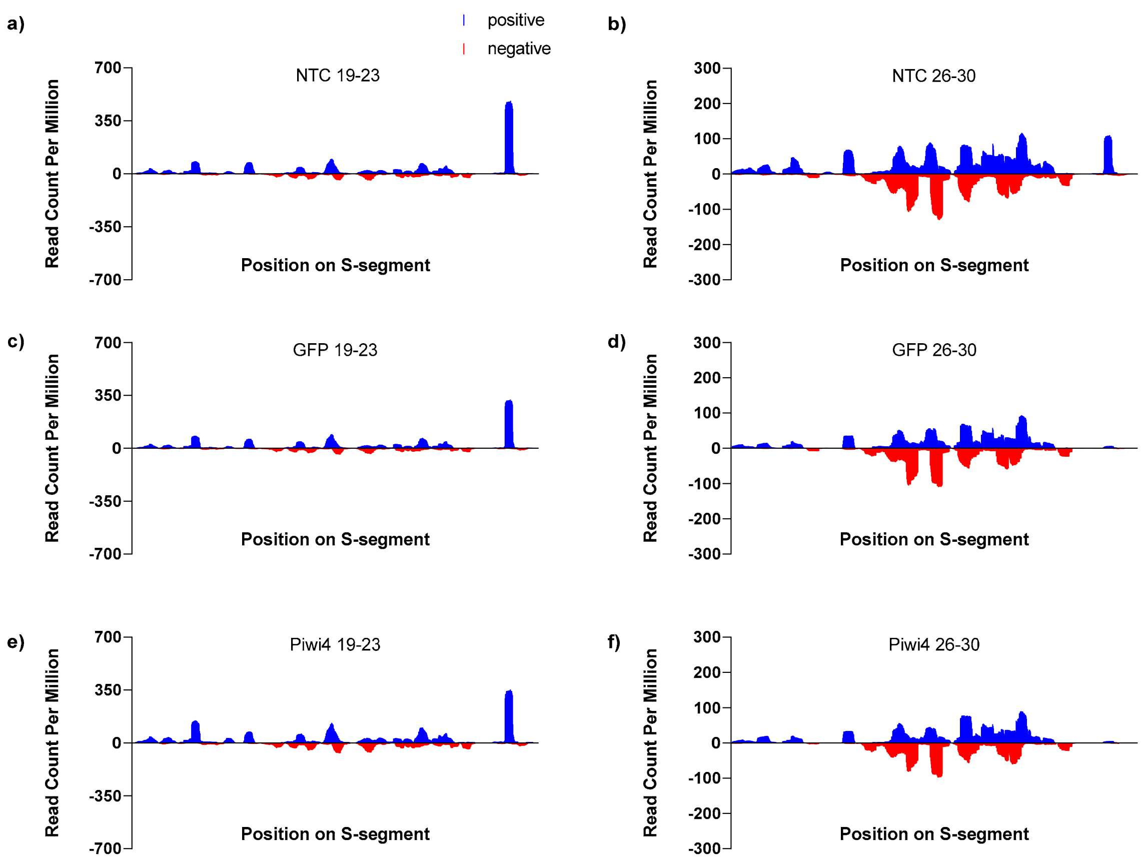

Supplemental Figure S4. sRNA reads mapped to LACV S-segment. 19-23nt (a,c,e) and specifically 26-30 nt (b,d,f) sRNA reads from LACV-infected Hsu cells were mapped along the LACV genome S segment. LACV-infected Hsu cells were treated with either no dsRNA (a,b), GFP dsRNA (c,d), or Piwi4 dsRNA (e,f).

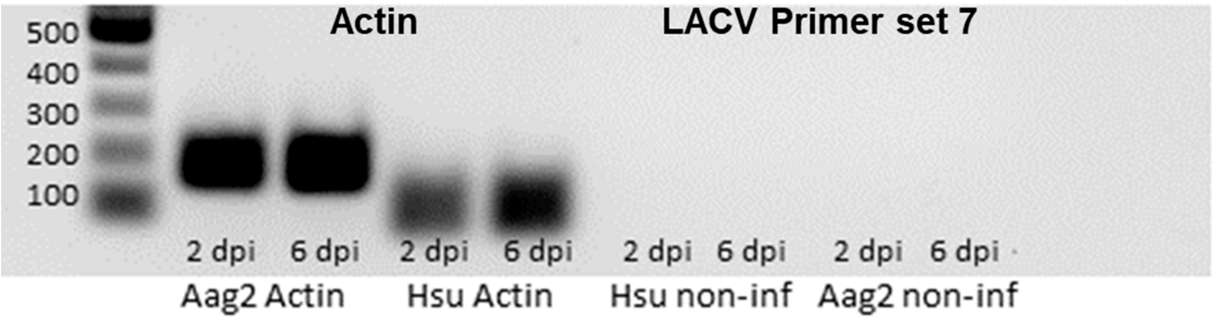

Supplemental Figure S5. LACV vDNA forms in Hsu and Aag2 cells. The housekeeping gene actin is seen at 2- and 6- dpi in Aag2 and Hsu cells, corresponding to DNA extractions from Figure 10. Hsu and Aag2 cells were left uninfected, and primer set 7 was used in PCR reactions, but no product was detected.

Supplemental Material S1: Culex tarsalis PIWI coding sequences.

>Piwi1_mRNA6102
atggtaaacaagctccgtagtgaacagctcacactgcagtccaagcacgaacgcaccggcgacgtttaccaaatcaagattgtcca
caccggaacggtggacatgaccaacgagacgggcattcaggtgctgaacttgatcctgcgtcgtgccatgggtggccttaacctgca
gctggtgggacgtaatctgtttgacgccgcagctaaaatcgcaatccgggagtatcagatcgaactgtggccggggtacgtgactagc
atccgacaacacgagcaggacattctcgtgtgctgcgaaattgcccacaagacgatgcgtatgcagacgtgttacgacattctgcgc
gagtgccagcgccacgatcgtaactacatggactcgttcaagcgtgccgtgctgggagttgtggtgttgaccgattacaacaacaaga
cctacacaatcaacgacgtttcgttcgataccaacccgcagagtacgttcgagaccaaaaacggccagacgtcctttgtggagtatta
ccagcagcggtacaacatccgcatccgggacacccagcagccgatgctgctgtcccgcgccaagaagcgggatctgcgtgcgggt
ggatgcgaattgatggccctggttccggaactgtgccgcgtgacgggactcaccgatcagatgcgctcggacttccgaatgatgaag
gctatgtcggatcatacgcggctgaacccggatcgtcgcatggagcgactcaacacgttcaataatcggctgcagacgtgccagga
aagtgcggaagtgttcaagatctgtcaaatggagctggatcgccgtttggttgatttgccgggacggttgctgccgcaagagttgatctttt
tctcgccgtctgcaaacggattcgcgtcgggtgagcaagctgactggtcgattcacttccggaacaatccaatgttttcaacgatccggc
tgaaccgctggttcttgattgtgccgcaacgggcgaacctgaaccgggatccacaaatgataatgtgcgtcgtgtcgaacaacaagg
ccgaccggtacacggcgatcaagaagaagtgctgcgtagatcgggcgattccaacgcaggtcatggtccaacgaacgatcacgcc
gaaggggggtaacgtgcgtacgctcatgtccgtagccacgaaggtcgtcatccagatgaactgcaagctcggtggtgtcccgtggaa
ggtcaagatcccgctgaacggactgatgacggtgggctttgacgtgtgtcacgacaccaaggacaagtccaagtcgtttaacaaggg
cgttccgaagtacttttccgcggtcagtcagcacacccacggcgaggagatttccaactatttgccgttaaacacggtgaaagcgctca
acgaataccggaaggaattcggcgaactgccgaagcggatcatcttctaccgggacggtgtcggcgagggccagctgcattacgtg
tacgaacacgaggtcaagtcgatcgtcgagaagctgagcgaagtgtacaaatcggcgggcaacgatcaggacgtgctgtttacgta
cttcatcgtgagcaagcggatcaacacgcgtttcttcgaacggcgcaacaatcccaagccgggaaccgtcgtcgatgacatcgtgac
gctgccggagcgaaccgacttttacctcgtgtcgcagtccgtgcgccagggcaccgtttcgccgaccgcttacaatgtcatctacgaca
cgtccaacctcaagattgaccacctgcagatgctgtcgtacaagcagtgccatctgtactacaactggtccggcacgacgcgcgttcc
cgccgtttgccagtacgcgcacaagttggccttcctggttggccagttcctgcaccagtcgccgagcaatctgctcgagaagaagctgt
acttcctctga

>Piwi4_mRNA2582
atgtctgatcgcgagcatgaccgcgaacgcccgagggcgcgtggatacaccgatagtcacgagggacgcggcagttccagtggg
cattcccgtccggcttggagtggtgagggtcgtgaccgtgccgagggacgtggaagccgggagcactcgcagagtcgtggcaatgg
gaatggtggagatcgtgaccgggaacggggaggtggtggtggtggacgacgggaaggtggtggaggtcgcgaggccggtggcc
gacgaggacgtcgtcgtacgtacggagatacactaagaacccgtggagcggatgccccttccaagcatggaaccaccggacagg
cactgcagctgcacagtaattatttcaagctgttgaagcacatcgagtggacactgtatcagtaccgagtggacttttcgccgcagtgcg
cgagttcccgcttgatgcagggtttgatcaacgaacacaaaaagatctttggcggtttcctgtttgatggcacccagctgtttatggtcaac
aagctgcgcagtgaccagctgacgttgcagtcccggcacgagcgtaccggcgatgtctatcagctgagaattatccacaccggatcg
gtggacatgaccaacgagaccggtattcaagtgctgaacttgattctgcgtcgtgcaatggctggtttgaaccttcagctggtcggacgt
aatctgttcgatgcggcggccaagatcgccatccgcgagtaccagattgagctgtggcccggttacattaccagtattcgtcagcacga
gcgggacattctcgtgtgctgtgaaattgctcacaaaacgatgcgtatgcagacctgttacgacatcctgcgcgattgccagaagcac
gaccgcaactacaaggactcgttcaagcgggccgtgctcggcgttgtcgtcctgaccgggtacaacaacaaaacctacaccatcaa
cgatgtcacgttcgagaccaccccggagagcaccttcgacaccaaggccggcaagacgtcctttgtggagtactacaagcaaaagt
acaacattcgcatccgagatccgcaccagcccatgctgctgtctcgtgccaagaagcgtgacctgcgtgccggtggcagcgagctg
atggctctggtgccggaactttgccagatgactggattgactgatcagatgagatcggatttcagaatgatgagagcgatggccgacc
atacccgtctcaatccggatcgtcgtatcgagcgattggagacattcaatcgccgactgcagacctcccccgagagcgttgaggtcttt
aagacctggcagatggagctggaccgacgtcttctggaagtccccggacgactgctgccacaggagatgatcttcttctcgaccacg
gcaaacggagtacaagccggtgagcaggccgattggacggcacacttccgtaacaacccgatgtttgcgacggcacggctgaatc
gttggtacttcatcgtacccaaccgatcgacccgcgaggccaacgatttttttcggttgcatgatccaggcggcccgcggaatgcgtttcg
agattagtaactgcgagactgtcaccattccggacgacaaccctgatacgtacgtgcgtacagtggacaacattctcaactaa

>Piwi5_mRNA13996
atggctgaacggcagcagcagggaaatcgtggccggggccgcgccagaggcttcgccggtgcgggccgcggtggtcaaggtca
gggaggccattaccaagggcagcagcctcctcagcaagctccgggtttccaagcaaaacgtgaggaaccttcgacgagccgtccc
cagcagctagcgagggctgaaggagccgtcggaggtcgcggagctatgcgcgggaaccggaaccttccggagctctttcgtacga
gaccagtgaacagtactaactcaaagcagggttcatcgggcattcagatcacgctgcagactaactactttaaggtgggacggaag
gataacgagagtatctttcaatatcgggttgatttcaacccgcccgtggagagttcgaggatgatgaatgcgctggttttcaatttgaagc
ctgtaatcggagggtatttatttgatgggacgcaacttttcacaaggcacagacttcgcagcgaggaggtcgagtatccgacgaagga
ccccaattcagggcaaaattacataattaagttgcggaaggtgggaaccattgacggagcgaccgagatggcgctcatgattttcaat
ttgatcaatcgtaaagcgatgaacgggctgaagttgacgctgattgggagaaactatttcgacgggcaggccaagatttccatccagc
agtacggaatcgatttatacccgggctatgtgaccagcatccgtcagcacgagcaagacgtgctgatgtgcgccgaactcacccatc
gagtcatgcgtacggacacgtgttacgcgatgttcaagacctgtctgaaccagggtggcaactggcgggacaactacaagcgcatg
gtgctgggaacggtcgtgatggccaactacggcaagaacaacacctacacggtcaacgacgtggagttcaacaccacaccggag
agttcgttcgaaaccagcaacggaaggattacctttttgcagtactttaaggaacgctacaacatcgtcatccgtgacccacgccaacc
gatgttggtatcgcgtgcgaaaccgcgcgacattcgtgccggaaaggcggaactgatctacctggtcccggagctgtttcgggcaac
cggactcaccgatgaaatgcgcaggaatttcaacttgatgcgaaccatgggggatcacacgcacctctcgccggacaagcgcatcg
agcggctggaaatgttcaaccggcggctgcagaactgtgaggaaagcgccgaagtgtttcgcttctggcaaaccgaactggatcgc
cgcctggttgaggtaacggggcgggtgctggctgaggaggagattctgttcaactacaatgagactagaaaagtacgtgccggccc
gcttgccgattggcagatagcgttccgcaaaaatcccatgttcttgtccgtcccgttgaagaaatggttctttatagtttcggctggggatca
gaacctgcaggatccgcagatgatcttctgcgtggtcagcaacgacaaggccgatcgttacgcggcgatcaagaagaagacttgcg
ttgagcgagcgattccgacgcaggtcatgaaagcgcggacgatcacgccgaagggtggtaacgtgcgaacgttgatgtcggtggct
acgaaggtcgccattcagatgaactgcaagctgggaggcattccgtgggtgttgaagaacccgctgtcgtcgatcatggtcattgggtt
cgatgtgtgtcacgattctcgcgacaagtccaaatctttcgggcaaaaacatcccaccttcttttcgaccgtcaaccatcactccagcggt
gaagaactgtccaactttatggcccagaacgttatcaaggcactgcactcgtacagggctgacttcggaggagcgctgcccaagcg
gatcatcatctaccgggatggagtaggcgagggccagttgaagtacgtgtacgaccacgaggtcggtgccgtcaaggaaaaattga
acatggtctacaaagattcgaagtcccaattgacgttcttcgttgtgaacaagcgcatcaacacgcgcctgttccaccagagacaaaa
tcccgttcccggaacaatcgtggatgacgtcattaccctgccagaaaggactgactttttcctggtgtcccaaagcgtccgccagggca
cggtctcgcccacctcgtacaacattctgctcgatgattccggcctggccatcgaccagctgcagatgtacaccttcaagcagacccat
ctgtactacaactggtccgggacggtaggggtcccggccgtgtgccagtacgcccacaagttggcctacctggctgggcagttcctgc
accaggcaccgaacaaccagatggagaagaagctgtactacttgtaa

>Piwi6_mRNA4897
atgtccgacgatcagcagcagccggagtgtagcagcagcagcggcggcggtcgaatccgggcacgtggcttcgtcggagcgtcac
gtggcggccgtaacggcaacggaagaccgagtggcggtggtggattgcccggatcggagcagcagcaccaccagcgtggccag
gtcggaagtttccagcaccgccagcagcagttccagaactacggcgccggtcgatcaccgggagggggaccacaggcgtcaggt
ggagctgggccagttccggttgaggaagtgtccgcgctggtgaaacagtcatcgccgagtgcgcaccgatcgtcggagcgggcgtt
ccagcggggaggagcggctgggggacgtggtgggatgcgcgggaatcggaacctgccggagattgtggtcaccaggaagcccg
gggacatgctgagcaagcatggcagcagcgggacgccgattttgctgcaggcgaactacttccgggtgattcgccacgaggatgag
cgtttgttcttgtaccgggtggattttacaccggcagtggaaacgaggagggcgcaaaactcgatcatgtacaatttgaagccccaaat
cgggggctatttgtttgacggaacgcaactatttacgcggaacaagctgtgcgacgaggagattcagtacgagaccaagtacaagg
cgaacaacgaagactacacgataaagctgcgcagagtcggcgtggtcgaagggacaaacgaggcagccttccaggtgtacaac
ctgatgaaccgcaaggccatgggaggcctcagattgcagctaatcgggcgcaacttcttcgacccggacgccaaggtcaacctcag
ccagtacaaaatcgacctctacccgggctacattaccagcatccggcagcacgagcaggacgtgctgatgtgcgccgaacttacgc
acaaggtgatgcgtacggacacgtgccacgacctgttcgagaagtgcatgatgaaccagcgcggcaacttacaggacaactacaa
gcgcgaggtgatcggcgcgaccgtgatgaccacctacggcagcaacaagatgtacgtggtgaacgatgtggactttagcctgtcgc
cggaaagcacgttcgagacgaggaatggcccgataacgttctttcagtacttccgcgatcggtacaacgtcacgatcaaggaccgg
ggccagccgatgttggtgtcgcgctcgaaggcgcgggatatccgggcgggaatgccggagctgatcatactggtgccggagctgtc
gcggatcacgggactgtcggacgagatgaggcgcgattttcacttgatgcgcggtctcgccgagcacacgcgcctcgcccccgatc

gtcgcatcgcccggctggaggcgttcaaccagcggctgcagttcagcaaggacagcgccgacacgttccgcttctggaagaccga
gctggaccgtcggcttgtggaggtgcaggggcgcgtcctagctccggagacgatcttcttccatccgcagtcggaccaatataaaatg
tcggccggagacaccgccgaatggcagatggcattccgggacaatccgatgtacctgacggtatcgctgaccaactgggcgatcgt
gattcctgggcatattcaacagagtgtgagccagttcttgggctgtttgtaccaggtgtcgcggcagatgcatttcctggttgatgagcctc
gctttgttccgatccagaacgattcaccggtggtctacatggagacgctgaatcagctcgttcagaaagatccccagctgatcatgtgc
attgtaaccaatgacaaagcggatcgctacgcggccatcaagaagaagtgctgcgtcgatcgagctgtgccgacgcaggtggtcaa
gtgcaagacgatcacgccgaagggtggcaatgcgcgcacgctcatgtccgtcgcaaccaagatagcgatccagctcaactgcaag
ctcggtggaatcccgtggatcgtcaagagtccgctgtcgtcggtgatgtgcctcggcttcgacgtttgccgggacaccaaagaccgga
acaaaacgtacggtgcgttggtagcgtcaatgtactacggcaagcaccgattccacccgagcttctactcgaccgtgaaccagcacg
ccaacggagcagagctgtccgattcgttggcgctgaacgtcgtcaaggcactgcgagcgtaccagcaaaagttcgaaaacaacct
gcccggccgtatcgttgtctaccgggacggagtcggtgacggagatctgcagtacgtgcacgagtacgaagtgggcgccatcaggg
acaagattgaagctatgtataaagcaagcggaatggaggccaagttctgcttcatagtcgtgagcaagcgtataaacacgcgcctgtt
caaccgcaagcaaaaccccgtgcccggaaccatcgtcgacgacgtgatcacgctgccagaacgcaacgacttctacctggtctcc
cagaacgtccgccagggcaccgtctcgccgacctcgtacaacgtgctgtacaacgattgcgggctggacgcggacaagctgcagg
tgtactcgttcaagcagacgcacatgtactacaactggtccggtacggtgcaggtgccggcggtgtgtcagtacgcgcacaagttggc
cgccctggccgggcagtttctgcatcaaccaccgagcacgtggttggagaggaagctgtactttttgtaa

>Piwi7_mRNA14507
atgcgttccggacgggacattccggatgtggtgcgaacgcggccggcggattgtcaggtgacgaagcacggaacttcgggacggc
agatccgcctgacggcgaactacttccgggtgatgcataccgacggggaggccatgttccagtaccgggtggactttgtgccggaga
ttgagagtgtgagggcgcggaaggcgttgatgcaccagctgaagccgaccctcggaccgatcctgttcgacggaggttccatctttat
gagccgggacaagacgcggagcgaggagagtgaaattacgacgaaggaaatgcagtcccagcaggagtacctggtgaagatc
aagaaggtcggagtggttgactggacgtcggagatggcgctgaccgtgctcaacctgatcaaccgacgcggcatgggaggacttcg
gctgcagcaaatcggacggaacttcttcgaccccaatgggaaggtccgactcgcggagttcgggctggaactctaccccggctacat
aaccagcatccggcagcacgagcagaacattctgatgtgcgcggaagtgacgcacaaggtcatgcgggaggacacctgctacgg
aatcctgcgcggctgcatggccaacggtggcaactggcaggccgactttacccgggcgatcctaggcactgtggtgatggcgatcta
cggccggaacaacacgtacaccgtgagcgatgtggagttcgccacgacgccggaacattcgttcgagacggcaaacgggagaat
gaccttcgtggagtactaccggagtcagtacaacatcgtcatccgggatccgcggcagccgatgctggtgtcgcgggccaggtcgcg
ggacgtacgggccggaaggccggagctgttgtacctggtgccggagctgatgcgcgcgaccgggttgacggatgagatgcggag
gaactttactttgatgcgcgccatggcggaccacacccggctgacgccggatcggcgcatgctgcggctagaggcgttcaaccagc
gcctgcacgacacccccgaaagtatcgaaatcttccgcttctggcagacggaactcgaccgccggctggtggaacttccgggccgc
gttctgcacccagagaagatcctgttcagcgagcagcagctgaatatgtgcgtcattccggacgactccgccgagtggcagatggcct
tccggaacaaccagatgtaccgctcggagcagctccagaactggttcctgatggttccgcgcaactgggtcgtcaaccgcgatccgc
agctgatcatgtgcatcgtgagcaacgataaagcggaccgctacgcggcgatcaagaccaagtgctgcgtcgaacgagcgatcgc
cacccaggtcatcaaggccaagacgatcacgccgaagggtggcaacgtgcgcacgttgatgtcggtggcgaccaaggtcgccatc
cagatgaactgcaagatcggcggaatcccgtggatcgtgacgaacccactgcgatcgatcatggtgatcggttacgacgtctgtcac
gacactcggaaccgatcgcgctccttctccatgaaacatccccgttacttctccaccgtcaaccaccactccggcggcgaagaagtgt
ccaacttcatggcccagaacgtggtcaaagctctgcgcgcctaccatgaagagttcaaggccctgcc
gaagcggatcatcatctacagggacggcgttggcgacggccaactcaagtacgtccacgagcacgaaatctcgtccatcctggcga
agctcaaactactcaacaaagacgatccaaccgccctcaagctgaccttcctggtggtcagcaagcgcatcaacacccgactcttcc
acgacaagcgcaacccggcgccgggaaccatcgtcgacgatgtcatcacgctcccggaacggaacgacttctacctggtgtcgca
gagcgtccgccagggcaccgtttcgcccacctcgtacaacgtgctgcacaacgagtccgggctgacggcggaccagctgcaggtgt
acacgtacaagcaaacgcacctgtactacaactggtccgggacggtcggcgtgccggcggtctgccagtacgcccacaagctggc
cgcactggccgggcagtatctgcaccaggcgccgcacagtgatctcgagaagaagctgtattatttgtaa

>Piwi8_mRNA5433
atgcgttccggaagggacatttcggcggtggtgcgaacgcgagcggcggactgtcaggtgacgaagcacggaacgtcgggacgg
cagatccgccttacggcgaactacttccgggtgatgcataccgacggggaggccatgttccagtaccgggtggactttgtgccggag
attgagagtgtgagggcgcggaaggcgttgatgcaccagctgaagccgaccctcggaccgatcctgttcgacggaggttccatcttta
tgagccgggacaagacgcggagcgaggagagtgaaattacgacgaaggaaatgcagtcccagcaggagtacctggtgaagatc
aagaaggtcggagtggttgactggacgtcggagatggcgctgaccgtgctcaacttgatcaaccgacgcggcatgggcggactccg
gctccaacaaatcggccggaacttcttcgaccccaacgggaaggtccgactcgcggagttcggcctggaactgtaccccggctacat
aaccagcatccggcagcacgagcagaacattctgatgtgcgcggaagtgacgcacaaagtcatgcgggaggacacctgctacgg
aatcctgcgcggctgcatggccaacggaggcaactggcaggccgactttacccgggcgatcctgggaactgtggtgatggcgatct
acggccggaacaacacgtacaccgtgagcgacgtggagttcgccacgacgccggaacattcgttcgagacggcaaccggaagg
atgaccttcgtggagtactaccggagtcagtacaacatcgtcatccgggatccgcggcagccgatgctggtgtcgcgggcccggtcg
cgggacgtacgggccggaaggccggagctgttgtatctggtgccggagctgatgcgggcgaccgggttgacggatgagatgcgga
ggaactttaccttgatgcgcgccatggcggaccacacccgactcactccggatcggcgcatgctgcggctggaggcgttcaaccagc
gcctgcacgacacccccgaaagtatcgaaatcttccgcttctggcagacggaactcgaccgccggctggtggaactgccgggccgc
gttctgcacccggagaagatcctgttcagcgagcagcagctgaatatcgtcattccggacgactctgccgagtggcagatggccttcc
ggaacaaccagatgtaccgctcggagcagctccagaactggttcctgatggttccgcgcaactgggtcgtcaaccgcgacccgcag
ctgatcatgtgcatcgtgagcaacgacaaagcggaccgctacgcggccatcaagaccaagtgctgtgtcgagcgagcgatcgcca
cccaggtcatcaaggccaagacgatcacgccgaagggtggcaacgtgcgcacgttgatgtccgttgcgaccaagggcgccagcc
agatgaactgcaagatcggcggaatcccgtggatcgtgacgaacccactgcgatcgatcatggtgatcggttacgacgtctgtcacg
acacccggaaccgatcgcgctccttctccatgaaacatccccgttacttctcgaccgtcaaccaccactccggcggcgaagaagtgtc
caacttcatggcccagaacgtggtcaaagcactgcgcgcctaccatgaagagttcaaggccctgccgaagcggatcatcatctacc
gggacggcgtcggcgacggccagctcaagtacgtccacgaacacgaaatctcgtccatcctagcgaagctcaaactgctcaacaa
agacgatccaaccgcgctcaagctgaccttcctcgtggtcagcaaacgcatcaacacccgactcttccacgacaagcgcaacccg
gcgccgggaaccatcgtcgacgatgtcattacgctcccggaacggaacgacttctacctggtgtcgcagagcgtccgccagggcac
cgtttcgcccacctcgtacaacgtgctgcacaacgagtccggactgacggcggaccagctgcaggtgtacacgtacaagcaaacg
cacctgtactacaactggtccgggacggtcggcgtgccggcggtctgccagtacgcccacaagctggccgcactggccgggcagta
cctgcaccaggcgccgcacagcgatctcgagaagaagctgtattacttgtaa

>Zucchini_mRNA10624
atgtcggtcatctccacgctgcggctgtcgtcggttccccgctggggctgggccgtaatcggtctcttcggtttcggtctgctcacggatctc
gtgtggaccggtgccgagcgctgggcgtcccggcggcgcgctcgccgccacacggaggccagcctgacggaggtgttcttcgcga
acgagcgcagcagccggccggacgacgccggcgttccgatggaccggttcacgtcggagcacgtccagcggatcgtcgactatct
ggaccgggcgcggctctccgtgtgcctcggcatgtacatcgtgacggtggacagcgtcggggatgcgctgattcgggcccggaacc
ggggcgttcgcgtgcgggtcatcggatgcgcctcgatggcgtacagttccggctcgcagatggtaaagttggctgaggaagatatccc
gatccggttcaacgacaagggcagtgcctacttgatgcaccacaagttctgcctgatcgacacggattggctgtgtcctcggtgctacc
acgcggaaagagtgcgccggctgggcttttgctgtgaccgtcccgcgaacgggcttccggcaaacggcagtgtgcttccggcaggc
cacgcaatggccagtggcagtggtgggggttagtggccaacggtggcggaagagcggcggtggttccgtgcaacctcagcgatc
gcggcagcggttgtgccggatgcagccgcgctttgcggcaggaaaaagagcgggcgttgatggcgcgggggaacgggatccgc
tgccggtgggggaatcatcatgaccgggtccacgaactggacgatgcaggcgctgtccggcaactgggacaacatcatcgtgac
gtcccggccggaagtggtcgtgccgttccaggtcgagttccagcggctgtgggcggtctttgcccggtcgcggccaatgatcgagcgg
ggagccgaggcgggggcgcagcagtag

>Ago3_mRNA4166
atgtcgtcccggttgaatctggtgcggacgttgctgagccagtccagctcatcggaggaccgcgcatccgggggaaccggaacgga
tccgtcgacggacaccggcttccagacgcgcaccccggagacgacgccaaactcgcagcgcaaagtcattggccgcggccagtc
ggctgttccgtcgcttaccacggccggaacgagcgggttggattcgtccgagtcggacaaccaggcggccagcgtggtgtccagcgt
tgcgccgacactttccggccgggggcgggctcagttcattcaagcgctgctgaagcagccgctggagtcgggtcgttcggttccgacc

ctggcggacgatgggacgagttcgatttcgtcggccccggccgtgtcgatgctgagtgcgggacggggtcggttcatgcagcagcta
atgaacagccgtgcggacgagccgccgagcgtgagcagcggaagcaagaccgagctgacggagcaaatgtcgcagattacgat
cgccgaaacggtgcagacggtggaaaagagtccggtcaagcggatgggcaacaagggaacgccggttcagctcatgacgaact
acattcggattgcgtgcgatccggatcgcggtctgtacgagtacgaggtgcgcttcagtccggaggtggactcgaaggtggcgcgttc
gaagtacatcaatcagcatcgcgaggtgattgggaacgcgaagacgttcgacggcgtgaagctgtttttgccgaagaagttgcccaa
cgcggaaaccattctgcagtcggaaaatgaagtcgatggccacacggtcacgattcggttgctgtacaagcgcaagcaacggatgt
cggaaaacattcagttctacaacatcctcttccagcgcattatgaaggtgttgaagatgatcgaaatgggtcgcaagaactttgatccgt
ccgcgccgaagctcatcccgcagcatcggttggaaatctggcccggttacgtgagcgccgttgacgagtacgaaggtgggctgatgt
tgaacctggacgtgtcgcatcgagtcctcctgcagactaccgtgctcgatcacatccgcacgatcgccaagacaaaccccagcgatt
ggaagaatctagtgaccaagtcgctgctcggagccgtcatcctgacccggtacaacaacaaaacgtaccgcatcgacgacatcatc
ttcgaccagaacccgacgatgacgttcatgtcgagcaaccggcagcagcagatctcgtacgtcgagtactacaagcagcagtacaa
catcacgattcacgacctgaagcaaccgctgctgattcaccgcaaggagcgccgcattgccggccaggacaaaccacaggagct
gatgatgtgcctcgttccggagattagctacctgaccggacttacggacgacatgcgcaacgacttcaaagtgatgcgtgacattgcg
gccttcacgcgggtctccccgaaccagcggctcaacagtatgcggcagttttgccacaacgtcaacgaaaacaaggaagcgcgtg
aaatcctcgaagtttggggccttaagctggacatggatccgctcatcatgcacggtcgtgccttcgaagaggaaaaagtcatcttcggc
aacgcccaagtcggcgtcggcaaagccggcgacttcaaccgggccgtcacgagcaacaacatgctgagcgccatcccgatccgc
aagtggctgctggtccacaccagcaaggacatgaaaatcgccaagtccttcatggactgcgtgcagcgcagctgccgccccatggg
catcgaagtcgacacgcccgccatcgaagtcctcccgacggacaaaaccgaactgtacgtgcagctgctgcgcaccaaaatccgc
caagacacgcaaatcgtcgtcatcatctgccccacgtctcgcgacgatcggtacgccgccatcaagcgaatctgctgcgcggaaatc
cccgtcccctcgcaggtcatcaacgcgcggacgctcgcgaacgaggccaaaaaccgcgccatcgtgcaaaagatcctgctccag
atgaactgcaagctgggcggaacgctgtggagcatcaaaatcccgttcaccaacgtgatgatcgccggtatcgacacgtaccacga
tccgaagcagaagagcaactcggtgtcggcgtttgtggcgtcgctgaacggagagtacacgcgctggtactcgcgcgcctgcatcca
gagcaagaaggaggagtttatgaacgggctgtgcgcctcgctggagaagtcgctgcggacgtacgagaagctgaacggggtgctg
ccgcagaagattatcatcttcagagacggcgtcggcgacggtcagctgcgaatgtgctcggagtacgagatcccgcagctgcagga
ggcgtgcaaactggtcgaaccgaactactgcccggagttggccttcatcgtggtgcagaagcgcatcaacactcgaatgttccggatt
gacggccaatccaacctggacaacccgaacccgggcaccgtgctggatcatactgtgacgcgacgcaaccacttcgactacttcct
ggtgccgcagtcggtccgccagggcagcgtttcgccgacgcattacatcgtcgtgtacaatcagcccgactacagcccggacgtgct
gcagcggctaagctacaagctgtgctatctgtactacaactggcccggaagtgtgcgagtgccggcctgctgccagtacgcccacaa
gatggcctacctgattgggcagtccgtgaagcgcaatccggacgaggtgctcaacgataagctgttctacctgtag

>**Sequence alignment of CtPiwi7 and CtPiwi8 using CLUSTAL O(1.2.4) multiple sequence alignment (97.3% nucleotide identity)**. Numbers (60, 120, etc.) indicate nucleotide position of the last nucleotide on the respective line.

```
Piwi7 60    atgcgttccggacgggacattccggatgtggtgcgaacgcggccggcggattgtcaggtg
Piwi8 60    atgcgttccggaagggacatttcggcggtggtgcgaacgcgagcggcggactgtcaggtg
            ************ ******** ***  **************  ******* *********
Piwi7 120   acgaagcacggaacttcgggacggcagatccgcctgacggcgaactacttccgggtgatg
Piwi8 120   acgaagcacggaacgtcgggacggcagatccgccttacggcgaactacttccgggtgatg
            ************** ******************** ************************
Piwi7 180   cataccgacggggaggccatgttccagtaccgggtggactttgtgccggagattgagagt
Piwi8 180   cataccgacggggaggccatgttccagtaccgggtggactttgtgccggagattgagagt
            ************************************************************
Piwi7 240   gtgagggcgcggaaggcgttgatgcaccagctgaagccgaccctcggaccgatcctgttc
Piwi8 140   gtgagggcgcggaaggcgttgatgcaccagctgaagccgaccctcggaccgatcctgttc
            ************************************************************
Piwi7 300   gacggaggttccatctttatgagccgggacaagacgcggagcgaggagagtgaaattacg
Piwi8 300   gacggaggttccatctttatgagccgggacaagacgcggagcgaggagagtgaaattacg
            ************************************************************
```

```
Piwi7 360  acgaaggaaatgcagtcccagcaggagtacctggtgaagatcaagaaggtcggagtggtt
Piwi8 360  acgaaggaaatgcagtcccagcaggagtacctggtgaagatcaagaaggtcggagtggtt
           ************************************************************
Piwi7 420  gactggacgtcggagatggcgctgaccgtgctcaacctgatcaaccgacgcggcatggga
Piwi8 420  gactggacgtcggagatggcgctgaccgtgctcaacttgatcaaccgacgcggcatgggc
           ************************************ ********************** 
Piwi7 480  ggacttcggctgcagcaaatcggacggaacttcttcgaccccaatgggaaggtccgactc
Piwi8 480  ggactccggctccaacaaatcggccggaacttcttcgaccccaacgggaaggtccgactc
           ***** ***** ** ******** ******************** ***************
Piwi7 540  gcggagttcgggctggaactctaccccggctacataaccagcatccggcagcacgagcag
Piwi8 540  gcggagttcggcctggaactgtaccccggctacataaccagcatccggcagcacgagcag
           *********** ******** ***************************************
Piwi7 600  aacattctgatgtgcgcggaagtgacgcacaaggtcatgcgggaggacacctgctacgga
Piwi8 600  aacattctgatgtgcgcggaagtgacgcacaaagtcatgcgggaggacacctgctacgga
           ******************************** ***************************
Piwi7 660  atcctgcgcggctgcatggccaacggtggcaactggcaggccgactttacccgggcgatc
Piwi8 660  atcctgcgcggctgcatggccaacggaggcaactggcaggccgactttacccgggcgatc
           ************************** *********************************
Piwi7 720  ctaggcactgtggtgatggcgatctacggccggaacaacacgtacaccgtgagcgatgtg
Piwi8 720  ctgggaactgtggtgatggcgatctacggccggaacaacacgtacaccgtgagcgacgtg
           ** ** ************************************************** ***
Piwi7 780  gagttcgccacgacgccggaacattcgttcgagacggcaaacgggagaatgaccttcgtg
Piwi8 780  gagttcgccacgacgccggaacattcgttcgagacggcaaccggaaggatgaccttcgtg
           **************************************** *** ** ************
Piwi7 840  gagtactaccggagtcagtacaacatcgtcatccgggatccgcggcagccgatgctggtg
Piwi8 840  gagtactaccggagtcagtacaacatcgtcatccgggatccgcggcagccgatgctggtg
           ************************************************************
Piwi7 900  tcgcgggccaggtcgcgggacgtacgggccggaaggccggagctgttgtacctggtgccg
Piwi8 900  tcgcgggcccggtcgcgggacgtacgggccggaaggccggagctgttgtatctggtgccg
           ********* **************************************** *********
Piwi7 960  gagctgatgcgcgcgaccgggttgacggatgagatgcggaggaactttacttgatgcgc
Piwi8 960  gagctgatgcgggcgaccgggttgacggatgagatgcggaggaactttaccttgatgcgc
           *********** ************************************** *********
Piwi7 1020 gccatggcggaccacacccggctgacgccggatcggcgcatgctgcggctagaggcgttc
Piwi8 1020 gccatggcggaccacacccgactcactccggatcggcgcatgctgcggctggaggcgttc
           ******************** ** ** *********************** *********
Piwi7 1080 aaccagcgcctgcacgacacccccgaaagtatcgaaatcttccgcttctggcagacggaa
Piwi8 1080 aaccagcgcctgcacgacacccccgaaagtatcgaaatcttccgcttctggcagacggaa
           ************************************************************
Piwi7 1140 ctcgaccgccggctggtggaacttccgggccgcgttctgcacccagagaagatcctgttc
Piwi8 1140 ctcgaccgccggctggtggaactgccgggccgcgttctgcacccggagaagatcctgttc
           *********************** ******************** ***************
Piwi7 1200 agcgagcagcagctgaatatgtgcgtcattccggacgactccgccgagtggcagatggcc
Piwi8 1197 agcgagcagcagctgaatat---cgtcattccggacgactctgccgagtggcagatggcc
           ********************   ****************** ******************
Piwi7 1260 ttccggaacaaccagatgtaccgctcggagcagctccagaactggttcctgatggttccg
Piwi8 1257 ttccggaacaaccagatgtaccgctcggagcagctccagaactggttcctgatggttccg
           ************************************************************
Piwi7 1320 cgcaactgggtcgtcaaccgcgatccgcagctgatcatgtgcatcgtgagcaacgataaa
Piwi8 1317 cgcaactgggtcgtcaaccgcgacccgcagctgatcatgtgcatcgtgagcaacgacaaa
           *********************** ******************************** ***
Piwi7 1380 gcggaccgctacgcggcgatcaagaccaagtgctgcgtcgaacgagcgatcgccacccag
Piwi8 1377 gcggaccgctacgcggccatcaagaccaagtgctgtgtcgagcgagcgatcgccacccag
           ***************** ***************** ***** ******************
```

```
Piwi7 1440 gtcatcaaggccaagacgatcacgccgaagggtggcaacgtgcgcacgttgatgtcggtg
Piwi8 1437 gtcatcaaggccaagacgatcacgccgaagggtggcaacgtgcgcacgttgatgtccgtt
           ******************************************************** ** 
Piwi7 1500 gcgaccaaggtcgccatccagatgaactgcaagatcggcggaatcccgtggatcgtgacg
Piwi8 1497 gcgaccaagggcgccagccagatgaactgcaagatcggcggaatcccgtggatcgtgacg
           ********** ***** *******************************************
Piwi7 1560 aacccactgcgatcgatcatggtgatcggttacgacgtctgtcacgacactcggaaccga
Piwi8 1557 aacccactgcgatcgatcatggtgatcggttacgacgtctgtcacgacaccggaaccga
           ************************************************** *********
Piwi7 1620 tcgcgctccttctccatgaaacatccccgttacttctccaccgtcaaccaccactccggc
Piwi8 1617 tcgcgctccttctccatgaaacatccccgttacttctcgaccgtcaaccaccactccggc
           ************************************** *********************
Piwi7 1680 ggcgaagaagtgtccaacttcatggcccagaacgtggtcaaagctctgcgcgcctaccat
Piwi8 1677 ggcgaagaagtgtccaacttcatggcccagaacgtggtcaaagcactgcgcgcctaccat
           ******************************************** ***************
Piwi7 1740 gaagagttcaaggccctgccgaagcggatcatcatctacagggacggcgttggcgacggc
Piwi8 1737 gaagagttcaaggccctgccgaagcggatcatcatctaccgggacggcgtcggcgacggc
           *************************************** ********** *********
Piwi7 1800 caactcaagtacgtccacgagcacgaaatctcgtccatcctggcgaagctcaaactactc
Piwi8 1797 cagctcaagtacgtccacgaacacgaaatctcgtccatcctagcgaagctcaaactgctc
           ** ***************** ******************** ************** ***
Piwi7 1860 aacaaagacgatccaaccgccctcaagctgaccttcctggtggtcagcaagcgcatcaac
Piwi8 1857 aacaaagacgatccaaccgcgctcaagctgaccttcctcgtggtcagcaaacgcatcaac
           ******************** ***************** *********** *********
Piwi7 1920 acccgactcttccacgacaagcgcaacccggcgccgggaaccatcgtcgacgatgtcatc
Piwi8 1917 acccgactcttccacgacaagcgcaacccggcgccgggaaccatcgtcgacgatgtcatt
           *********************************************************** 
Piwi7 1980 acgctcccggaacggaacgacttctacctggtgtcgcagagcgtccgccagggcaccgtt
Piwi8 1977 acgctcccggaacggaacgacttctacctggtgtcgcagagcgtccgccagggcaccgtt
           ************************************************************
Piwi7 2040 tcgcccacctcgtacaacgtgctgcacaacgagtccgggctgacggcggaccagctgcag
Piwi8 2037 tcgcccacctcgtacaacgtgctgcacaacgagtccggactgacggcggaccagctgcag
           ************************************** *********************
Piwi7 2100 gtgtacacgtacaagcaaacgcacctgtactacaactggtccgggacggtcggcgtgccg
Piwi8 2097 gtgtacacgtacaagcaaacgcacctgtactacaactggtccgggacggtcggcgtgccg
           ************************************************************
Piwi7 2160 gcggtctgccagtacgcccacaagctggccgcactggccgggcagtatctgcaccaggcg
Piwi8 2157 gcggtctgccagtacgcccacaagctggccgcactggccgggcagtacctgcaccaggcg
           *********************************************** ************
Piwi7 2199 ccgcacagtgatctcgagaagaagctgtattattgtaa
Piwi8 2196 ccgcacagcgatctcgagaagaagctgtattacttgtaa
           ******** *********************** ******
```
